# Transient Receptor Potential Vanilloid 2 Regulates Myocardial Response to Exercise

**DOI:** 10.1371/journal.pone.0136901

**Published:** 2015-09-10

**Authors:** Mindi Naticchioni, Rajiv Karani, Margaret A. Smith, Evan Onusko, Nathan Robbins, Min Jiang, Tatiana Radzyukevich, Logan Fulford, Xu Gao, Ryan Apel, Judith Heiny, Jack Rubinstein, Sheryl E. Koch

**Affiliations:** 1 Department of Internal Medicine, Division of Cardiovascular Health & Disease, University of Cincinnati College of Medicine, Cincinnati, Ohio, United States of America; 2 Department of Molecular and Cellular Physiology, University of Cincinnati College of Medicine, Cincinnati, Ohio, United States of America; University of Debrecen, HUNGARY

## Abstract

The myocardial response to exercise is an adaptive mechanism that permits the heart to maintain cardiac output via improved cardiac function and development of hypertrophy. There are many overlapping mechanisms via which this occurs with calcium handling being a crucial component of this process. Our laboratory has previously found that the stretch sensitive TRPV2 channels are active regulators of calcium handling and cardiac function under baseline conditions based on our observations that TRPV2-KO mice have impaired cardiac function at baseline. The focus of this study was to determine the cardiac function of TRPV2-KO mice under exercise conditions. We measured skeletal muscle at baseline in WT and TRPV2-KO mice and subjected them to various exercise protocols and measured the cardiac response using echocardiography and molecular markers. Our results demonstrate that the TRPV2-KO mouse did not tolerate forced exercise although they became increasingly exercise tolerant with voluntary exercise. This occurs as the cardiac function deteriorates further with exercise. Thus, our conclusion is that TRPV2-KO mice have impaired cardiac functional response to exercise.

## Introduction

The myocardial response to pressure overload is a well-established adaptive mechanism to physiologic stress. Typically, the exercise-induced changes associated with alterations in the size and shape of the heart is collectively referred to as ventricular remodeling [1]. The development of physiologic hypertrophy consists broadly of increased cardiac size, improved cardiac function, development of myocyte hypertrophy (without fibrosis), relatively unchanged fetal gene profile and slightly increased expression of calcium handling proteins [2,3].

The endurance (isotonic) exercise-induced changes in the heart for humans and mice have been determined to be LV dilation and eccentric hypertrophy [1,4,5]. The Transient Receptor Potential Vanilloid 2 (TRPV2) channel has been shown to play a critical role in eccentric hypertrophy in dystrophic-deficient mice [6].

Our laboratory has recently found that the TRPV2 channels are expressed in the murine myocytes and regulate calcium homeostasis and contractility on a beat-to-beat basis [7]. Our laboratory has also found that mice lacking TRPV2 (TRPV2-KO) have impaired cardiac function at baseline, though no significant vascular phenotype [7]. TRPV2 has been previously described as a stretch mediated channel [8] and a regulator of calcium homeostasis in other cell types [9]. It is also known to play a crucial role in the development of cardiomyopathy [10,11] and its deletion results in impaired systolic and diastolic function [7], though its role under conditions of physiologic stress on the heart has yet to be established. Based on these observations, we hypothesized that TRPV2 plays a critical role in the cardiac response to stress and in the development of physiologic hypertrophy and therefore, ablation of this channel will impair the cardiac response to exercise. Hence, we sought to examine the molecular interactions between TRPV2 dependent calcium modulation and other calcium handling proteins after physiologic stress (exercise) [12,13].

Herein, we present evidence that the impaired cardiac function associated with TRPV2 deficiency blunts the normal myocardial response to voluntary and forced exercise and markedly reduces exercise tolerance. Therefore, we postulate that TRPV2 is a crucial mediator of myocardial response to stress.

## Methods

### Ethics Statement

All animal procedures were performed with the approval of the Institutional Animal Care and Use Committee (IACUC) of the University of Cincinnati and in accordance with the Eighth Edition of the *Guide for the Care and Use of Laboratory Animals* [14]. All wild type (WT) and TRPV2-KO mice (B6129FS2/JF2 background) were males 8–20 weeks of age. Breeding pairs for the TRPV2-KO mice were provided by Dr. M. Caterina at John’s Hopkins, Baltimore, MD [15].

### Quantitative RT-PCR

Total RNA was isolated (Qiazol method, Qiagen, Venlo, Limburg, Netherlands) and cDNA synthesized (High Capacity RNA-to-cDNA kit; Applied Biosystems, Carlsbad, CA) per manufacturer’s instructions. Expression levels of TRPV2 and brain natriuretic peptide (BNP) were determined using the primer sets listed in Table 1. All PCR experiments were performed as previously described [16].

**Table 1:** Primer sequences for RT-PCR experiments.

		Primer Sequence		
Protein	Abbreviation	Sense	Antisense	Product size
Transient Receptor Potential Vanilloid type 2	TRPV2	CTACTGCTCAACATGCTC	CTCATCAGGTATACCATCC	197bp
TRPV2 N-terminus	N-term	GGCTGGAGACGTCCGATGGAG	CGGTAGTTGAGATTCACTTTAATC	138bp
TRPV2 Segment 1-Segment 3	S1-S3	CCATAGTTGCCTACCACC	GAGATCCAGATGAACAGGC	175bp
TRPV2 Segment 4-Segment 6	S4-S6	GGCATCTACAGTGTCATG	GCTCCAGCTGTCAGTGGC	389bp
TRPV2 C-terminus	C-tail	GTAAACTGGGCTGCATGG	GTCTTCCTCTGAGGCACTG	107bp
Brain Natriuretic Peptide	BNP	AAGTCCTAGCCAGTCTCCAGA	GAGCTGTCTCTGGGCCATTTC	91p
GAPDH	GAPDH	CATGGCCTTCCGTGTTCCTA	CCTGCTTCACCACCTTCTTGATG	105bp

### Western blot analysis

Protein was isolated from heart samples, as previously described [17]. Briefly, heart samples were homogenized in the following buffer: 20mM HEPES (pH 8.0), 150mM NaCl, 1% sodium deoxycholate, 1% SDS, protease inhibitor cocktail (P8340; Sigma Aldrich, St. Louis, MO) and phosphatase inhibitor cocktail (EMD, Merck, Darmstadt, Germany) and centrifuged at 100,000xg for 20 minutes. Protein concentration and western blot analysis were conducted as previously described [16]. 100μg of protein was loaded on a 10% gel for TRPV2 (AB5398, Chemicon, Temecula, TX). Proteins bands were visualized using Western Lightning reagents (Perkin Elmer, KY) and the FluorChemE (ProteinSimple, CA). The densitometry of the bands was determined using AlphaEase software (ProteinSimple formally Alpha Innotech, CA) and normalized to GAPDH for loading control.

### Histopathology

Tissues were fixed overnight in 4% paraformaldehyde and further dehydrated with sequential 30%, 50% and 70% ethanol before final processing using Excelsior Tissue Processor according to manufacturer’s instructions (Thermo Scientific). Tissues were then embedded in paraffin and sectioned (5 μm) for H&E staining and immunohistochemistry as previously described [7,18]. Antibodies were as follows: Anti-vanilloid receptor like protein 1 (TRPV2; Millipore, AB5398, 1:1000).

### Treadmill (Forced exercise)

WT and TRPV2-KO mice, 8–10 weeks old, were subjected to forced running on an Accupacer treadmill (Omnitech Electronics Inc, Columbus, OH) based on previously published protocols [19]. The mice were subjected to four different treadmill protocols during the course of this study. First, to familiarize the mice to the treadmill, they were run for 3–5 days at 10–14 m/min (for 10 minutes) [19]. Second, the mice were given a physical capacity test to verify that they were capable of running the remaining protocols. For the physical capacity test, the treadmill was set to 14 m/min and the shock strength was set to 1.5 mA. The speed was increased by 2 m/min every 20 seconds until failure. Third, the mice underwent daily conditioning for 4 weeks (Table 2). Lastly, the mice were subjected to endurance testing once a week. Following the daily conditioning, the mice were given a 10 minute break with the shock still on. For the endurance test, the mice were run at 22 m/min (medium speed) and the shock strength was set to 2.0 mA. The time of each mouse’s failure was recorded. The mice were euthanized after 4 weeks of forced exercise. Shocks were only counted when one of the following criteria was met: the mouse fell onto the shock bars, any part of the mouse touched the metal shock bars (exception: the mouse’s tail) or the mouse noticeably jumped from a shock. Failure was defined as the mouse can no longer run, the mouse does not respond to a shock or the mouse has reached total 50 shocks for the day.

**Table 2:** Daily Treadmill protocol.

Daily Conditioning	Speed (m/min)	Time (minutes)	Shock Strength (mA)
**Warm-Up**	8	5	2.0
**Interval 1**	14	4	2.0
**Active Rest 1**	8	2	2.0
**Interval 2**	16	4	2.0
**Active Rest 2**	8	2	2.0
**Interval 3**	18	4	2.0
**Active Rest 3**	8	2	2.0
**Interval 4**	20	4	2.0
**Cool Down**	8	5	2.0

### Wheel (Voluntary exercise)

WT and TRPV2-KO mice, 8–10 weeks old, were placed in special cages large enough to house an 11.4 cm pet wheel (Super Pet, Elk Grove Village, IL) to test voluntary running [19,20,21]. The wheels were connected to a SigmaSport (TARGA, St. Charles, IL) bike counter in order to monitor the total time spent running, the distance ran and the average and maximum velocity for each individual mouse. Each counter was checked daily and values for all the parameters were recorded and reset. The mice were euthanized after 8 weeks of voluntary exercise.

### Skeletal muscle experiments

Contractility measurements were performed on isolated EDL muscles as described previously [22,23]. Briefly, the EDL was surgically removed and mounted horizontally in a chamber between two platinum plate electrodes for field stimulation. One tendon was tied to a plastic ring by surgical silk and fixed to a stainless steel post, and the opposite tendon was fixed by surgical silk to the lever of an isometric force transducer. Muscles were incubated in physiological solution containing (mM): 118 NaCl, 4.7 KCl, 25 NaHCO3, 2.5 CaCl2, 1.2 MgSO4, 1.2 NaH2PO4, 0.026 EDTA, 11 glucose, equilibrated with 95% O2-5% CO2 at room temperature (22°C), pH 7.4. The bathing solution was continuously perfused through the chamber (chamber volume: 1 ml) at a speed of 1.5 ml/min and the chamber was covered to maintain a constant environment. The muscle was stimulated by rectangular pulses of 0.5 ms duration. Supramaximal voltage and optimal muscle length were determined by series of 0.5 Hz pulses to elicit maximal isometric twitch contractions. For tetanic stimulation, trains of pulses 40, 70, 80, 100 and 125 Hz and 400 ms duration were applied. Fatiguing stimulation consisted of a series of 40 Hz trains of 750 ms duration repeated every 7.7 or every 3 s until force declined to 50% of maximum tetanic force, or until 2 min had elapsed. The overall experimental sequence was: 1) determine stimulus amplitude and optimal rest length (Lo); 2) record reference tetanic force at 40 Hz, 400 ms; 3) apply single stimuli every 3 min to achieve constant twitch force; 4) apply tetanic stimuli of 40–125 Hz/400 ms every 3 min to determine peak tetanic force at each frequency and maximum tetanic force; 5) repeat reference tetanus (end); 6) apply fatiguing stimulation until force declines; and 7) measure Lo and muscle weight. This protocol provided multiple contractile measurements from the same muscle with high reproducibility in a typical recording period of 30 min. Force data were recorded digitally (BioPac System, Goleta, CA) at a sampling frequency of 500 Hz. Specific force (mN/mm2) was expressed relative to cross-sectional area using the method of Brooks et al [24].

### Echocardiography

Echocardiography measurements were taken at baseline, i.e. prior to surgery or exercise, and weekly thereafter for all groups. All echocardiographic studies were performed as previously described [16]. Briefly, mice were anesthetized with isoflurane and placed on the heated stage of the Vevo 2100. Parasternal long axis (PSLAX) and short axis (SAX) images were recorded and then analyzed on a separate work station with VevoStrain software (Vevo 2100, v1.6, Visualsonic, Toronto, Canada). From the M-mode images, left ventricular cavity size for systole and diastole (LV Vol;s and LV Vol;d, respectively) and the diastolic diameter were measured. The rest of the echocardiographic calculations; Ejection Fraction (%EF), Fractional Shortening (FS), Stroke volume (SV) and Cardiac Output (CO), were obtained using the Vevo software. The change in SV, EF and LV diastolic volume was determined by subtracting the baseline measurement from the end point echocardiography measurement for each mouse (4 weeks for forced and 8 weeks for voluntary). In addition, as a measure of the development of hypertrophy, we determined the intraventricular septal wall width during diastole (IVS;d).

### Data Analysis

Statistical analysis was performed by paired and unpaired Student’s *t*-test and analysis of variance (ANOVA) using either a single factor within-subjects design, or a two-factor mixed design with repeated measures as appropriate. Where significance was indicated, post-hoc testing was performed using the Holm-Sidak method for comparing individual means and correcting for family-wise error (SigmaPlot v.13.0, Systat Software, Inc., San Jose, CA). Data are presented as means ± S.E.M., and differences were regarded as significant at P ≤ 0.05.

## Results

### RT PCR of different segments of TRPV2

**Fig 1:**
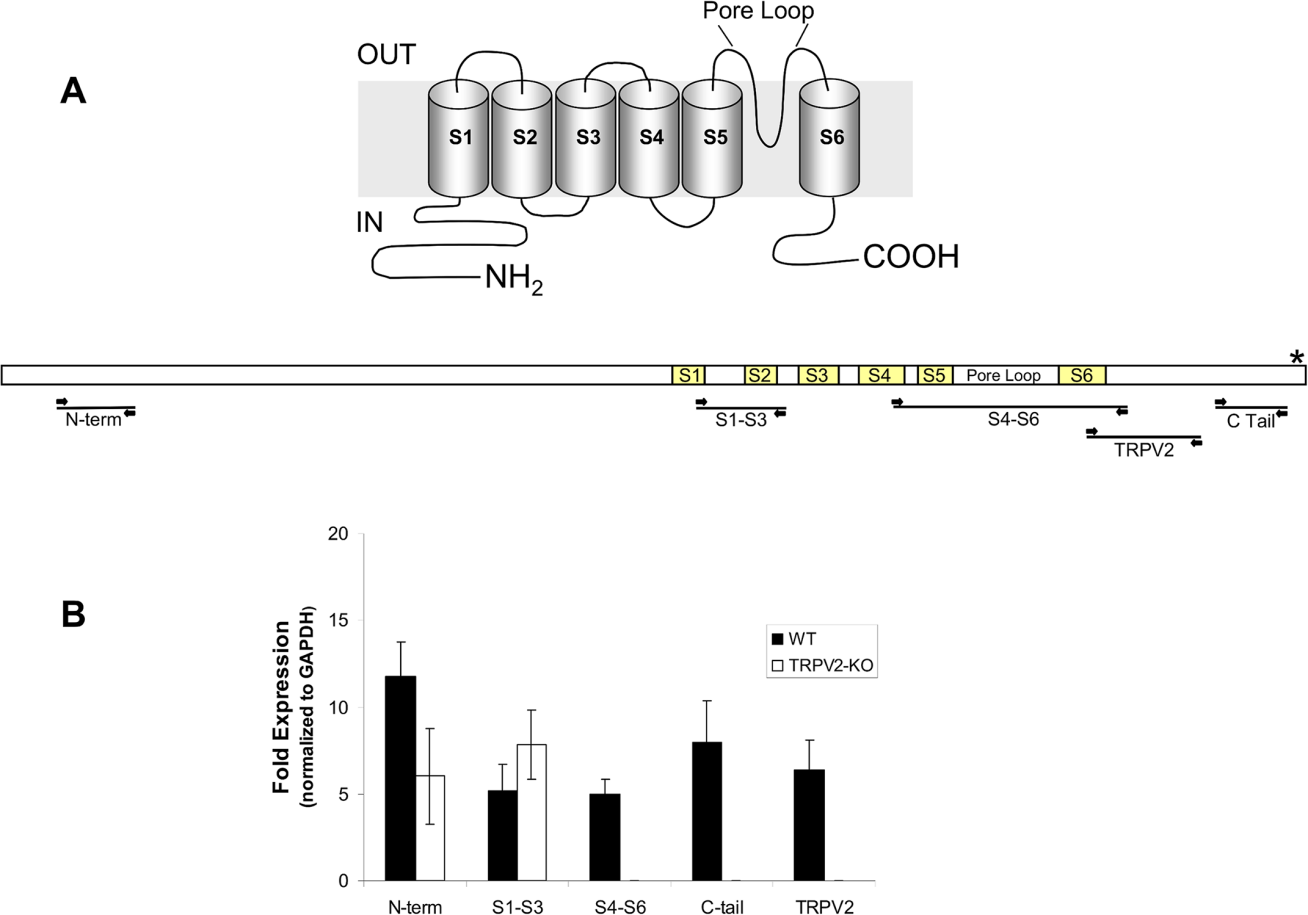
RT-PCR analysis of the TRPV2 protein in WT and TRPV2-KO mice. (A) Schematic of the TRPV2 channel, including RT-PCR primer locations (*indicates TRPV2 antibody binding site). (B) RT-PCR expression results from the primer pairs (see Table 1).

TRPV2-KO is a functional knockout, as it is missing the pore loop which contains the active part of the channel, namely the Ca^2+^ binding site (Schematic diagram of TRPV2 and the primer locations in Fig 1A). RT-PCR using primers from various regions of the TRPV2 gene indicate that the N-terminus and S1-S3 regions are still present in the TRPV2-KO mouse, further, we show that expression of the deleted portion of the channel is absent in the TRPV2-KO mouse (Fig 1B).

### Western blot and IHC of TRPV2

**Fig 2:**
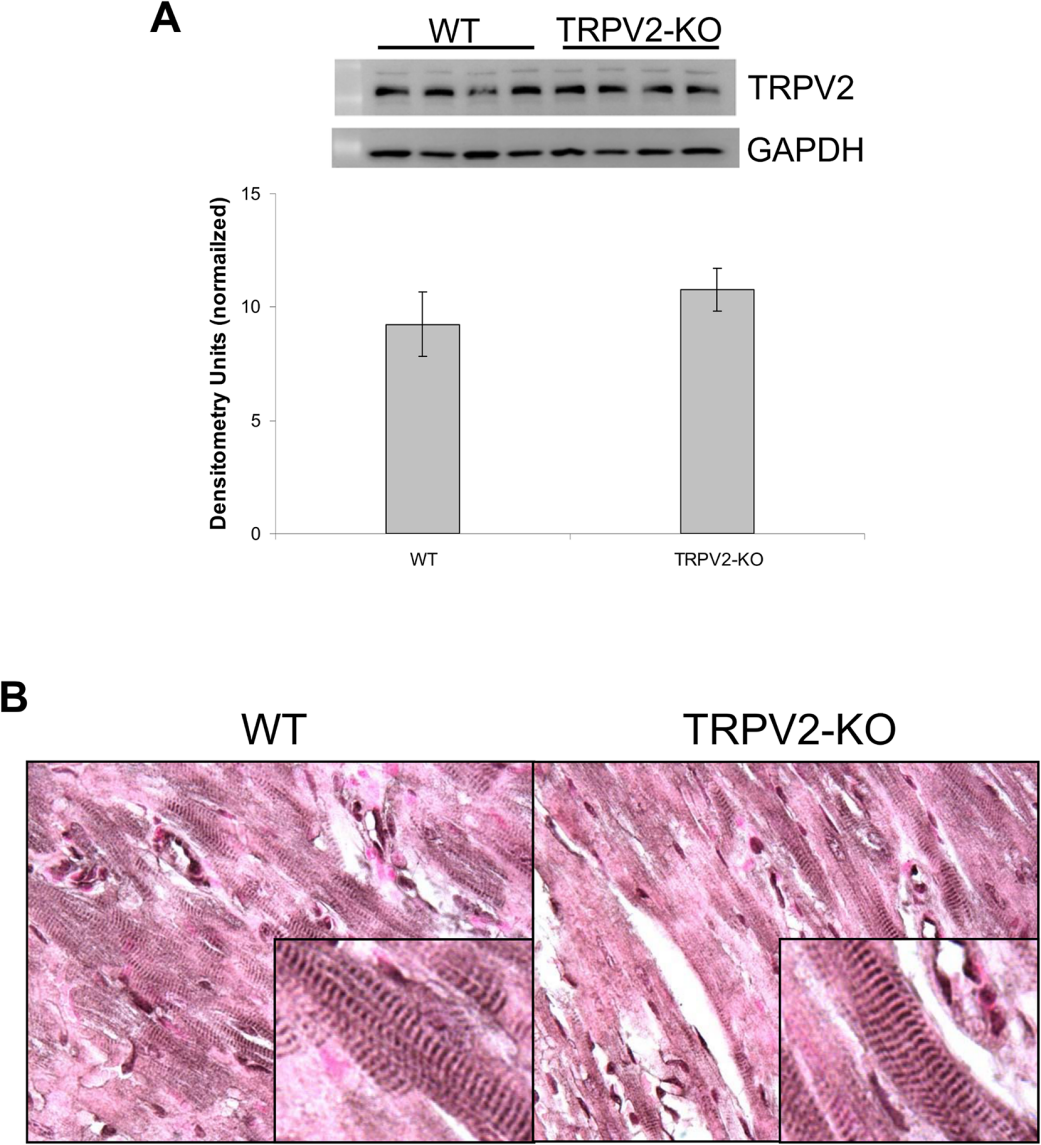
TRPV2 protein expression analysis. (A) Western blot and (B) IHC staining in WT and TRPV2-KO mouse hearts.

As described above, the TRPV2-KO is a functional knockout and the majority of the protein is still expressed, though it does not function as a Ca^2+^ channel due to the lack of a pore loop. An antibody to the last 18 amino acids of the C-tail still recognizes the modified TRPV2 protein via western blot (Fig 2A) and IHC (Fig 2B). IHC analysis showed a similar protein localization WT and TRPV2-KO mouse hearts (Fig 2B, insets).

### In vivo studies

#### TRPV2 null mice have impaired exercise capacity

**Fig 3:**
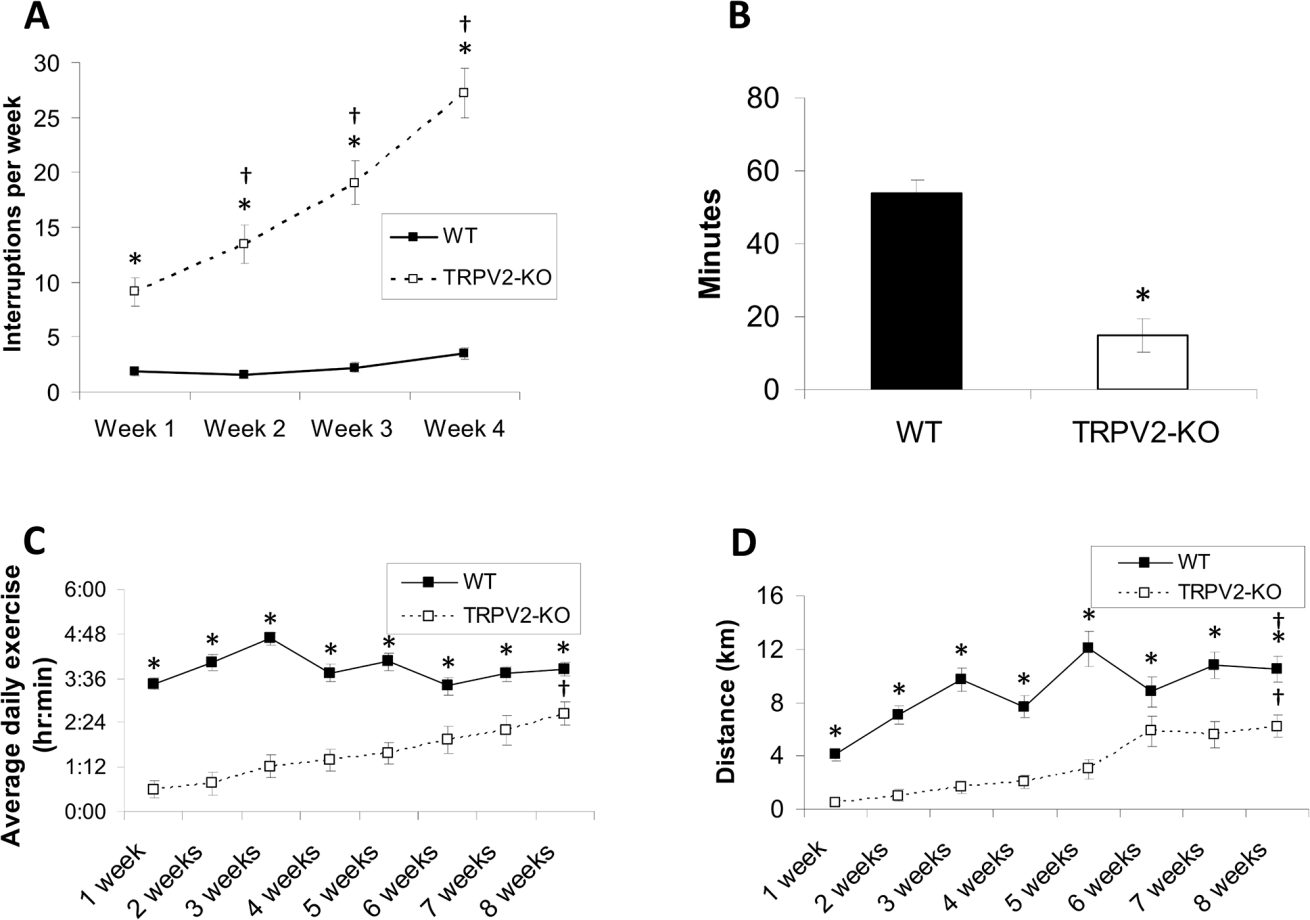
Exercise response of the WT and TRPV2-KO mice. (A) Number of interruptions in daily running of WT and TRPV2-KO mice (n = 5 for both groups) and (B) total time to failure during endurance runs. (C) Average daily exercise time and (D) distance in WT and TRPV2-KO mice. (*P<0.05, compared to WT and †P<0.05, compared to baseline).

In light of the decreased systolic function of TRPV2-KO mice under baseline conditions, we tested their exercise capacity under duress with a forced exercise protocol as previously described [19]. The TRPV2-KO mice were able to exercise for significantly less time than their WT counterparts, based on the increasing number of interruptions during the daily “high intensity” exercise protocol (Table 2, Fig 3A), and in fact demonstrated a decreased capacity for exercise over the 4 week experiment. In addition, the amount of time the TRPV2-KO mice were able to exercise on the weekly endurance protocol (Fig 3B) was significantly less than that of the WT mice.

Due to the fact that the TRPV2-KO mice were unable to exercise with a forced exercise protocol, 6 WT and 6 TRPV2-KO mice were subjected to a voluntary exercise protocol as previously described [20]. During the first five weeks of exercise, the TRPV2-KO mice had markedly impaired exercise ability as measured by exercise time and weekly distance (Fig 3C and 3D). Over the 8 week period, all mice were able to improve their exercise capacity, although the time and distance that the TRPV2-KO mice were able to exercise was considerably lower than the WT.

#### TRPV2 null mice do not have a skeletal muscle phenotype

**Fig 4:**
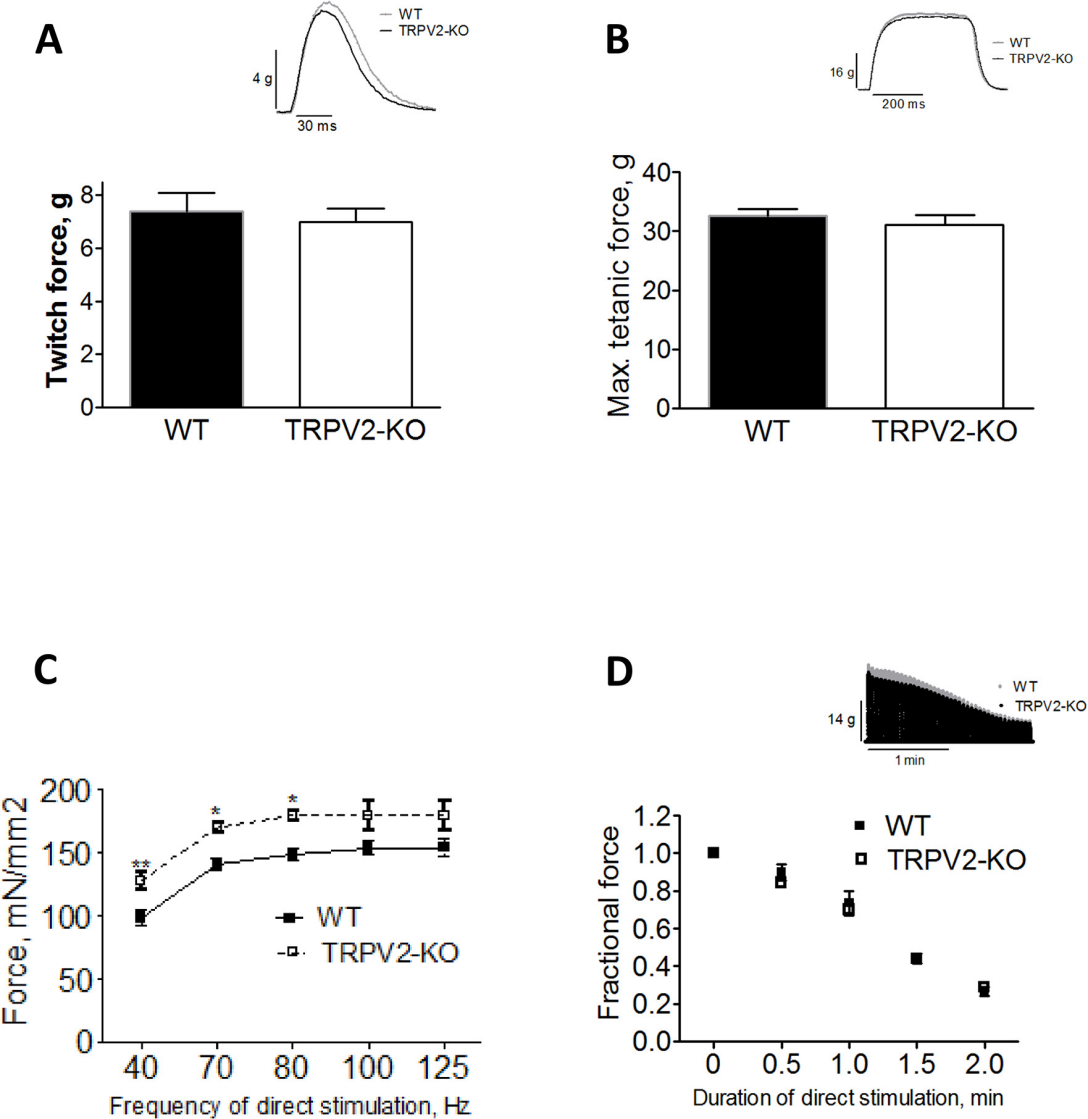
Contractility and fatigability of isolated skeletal muscles from TRPV2-KO and WT mice. (A) Mean twitch force produced by the EDL muscle of WT and TRPV2-KO mice. *Inset*: representative recording of twitch force. (B) Maximum tetanic force produced by the same muscles in response to a single tetanus of 80 Hz, 400 ms duration. *Inset*: representative recording of maximum tetanic force. (C) Specific Force vs. stimulation frequency of WT (ο) and TRPV2-KO (•) EDL (**P<0.01, *P<0.05). (D) Force vs. duration of stimulation for WT (ο) and TRPV2-KO (•) EDL muscles subject to fatiguing stimulation (40 Hz/750 ms tetani repeated every 3 sec for 2 min). *Inset*: representative force recordings. (n = 5–6 muscles from 4 mice of each genotype.) TRPV2-KO skeletal muscles are not significantly different from WT.

To establish if the exercise impairment in TRPV2-KO mice was secondary to impaired myocardial function, or to a possible skeletal muscle abnormality, contractility measurements of isolated extensor digitorum longus (EDL) muscles was performed as previously described [23]. The EDL is the most relevant muscle group used in treadmill running. The mouse EDL is a fast-twitch muscle and has a fiber type composition of 49% FG (Type IIA, fast twitch oxidative) and 51% FOG (Type IIX, fast twitch glycolytic). This fiber type composition is representative of the hind limb muscles used in running [25]. EDL weight was lower in TRPV2-KO compared to WT mice (8.4±0.3 mg (n = 6) and 11.3±0.8 mg (n = 5), respectively, P<0.001). Twitch (Fig 4A) and tetanic (Fig 4B) force were not significantly different between genotypes. Both genotypes showed similar force-frequency dependence, with tetanic force reaching a maximal value at stimulation frequencies above 80 Hz (Fig 4C). Specific force normalized to cross sectional area was significantly greater in the TRPV2-KO compared to WT at 80 Hz stimulation (Fig 4C; 180.2 ±4.0 (n = 3) and 149.3±4.6 (n = 4), respectively, but did not reach significance at 100 Hz, (179.9±11.1 (n = 3) and 154.2 ±5.7 (n = 4), respectively, P>0.05). These results indicate that the skeletal muscles of TRPV2-KO mice are able to produce twitch and tetanic forces that are comparable to or greater than WT, and to increase force with increasing frequency in the expected manner.

Skeletal muscles maintain contractility during sustained use and therefore the ability of TRPV2-KO mice to perform during (running) exercise requires that the intrinsic mechanisms are not impaired. Thus, the fatigability of EDL muscles from WT and TRPV2-KO mice was compared using both a mild fatigue-inducing protocol [23] (repeated 40 Hz/750 ms tetanic stimulation applied every 7.7 sec), and a more intense protocol (same tetanic stimulation applied every 3 sec). WT and TRPV2-KO mice showed no difference in fatigability with either the mild (data not shown) or the more intense protocol (Fig 4D). For the intense protocol, the time for force to decline to 50% was 82±3 s (n = 5) for WT and 83±2 s (n = 5) for TRPV2-KO (P>0.05). Therefore, the TRPV2-KO mice do not show any impairment in force production or fatigability of the skeletal muscles that could account for their dramatically reduced exercise capacity.

#### TRPV2 null mice have impaired cardiac response to exercise

**Fig 5:**
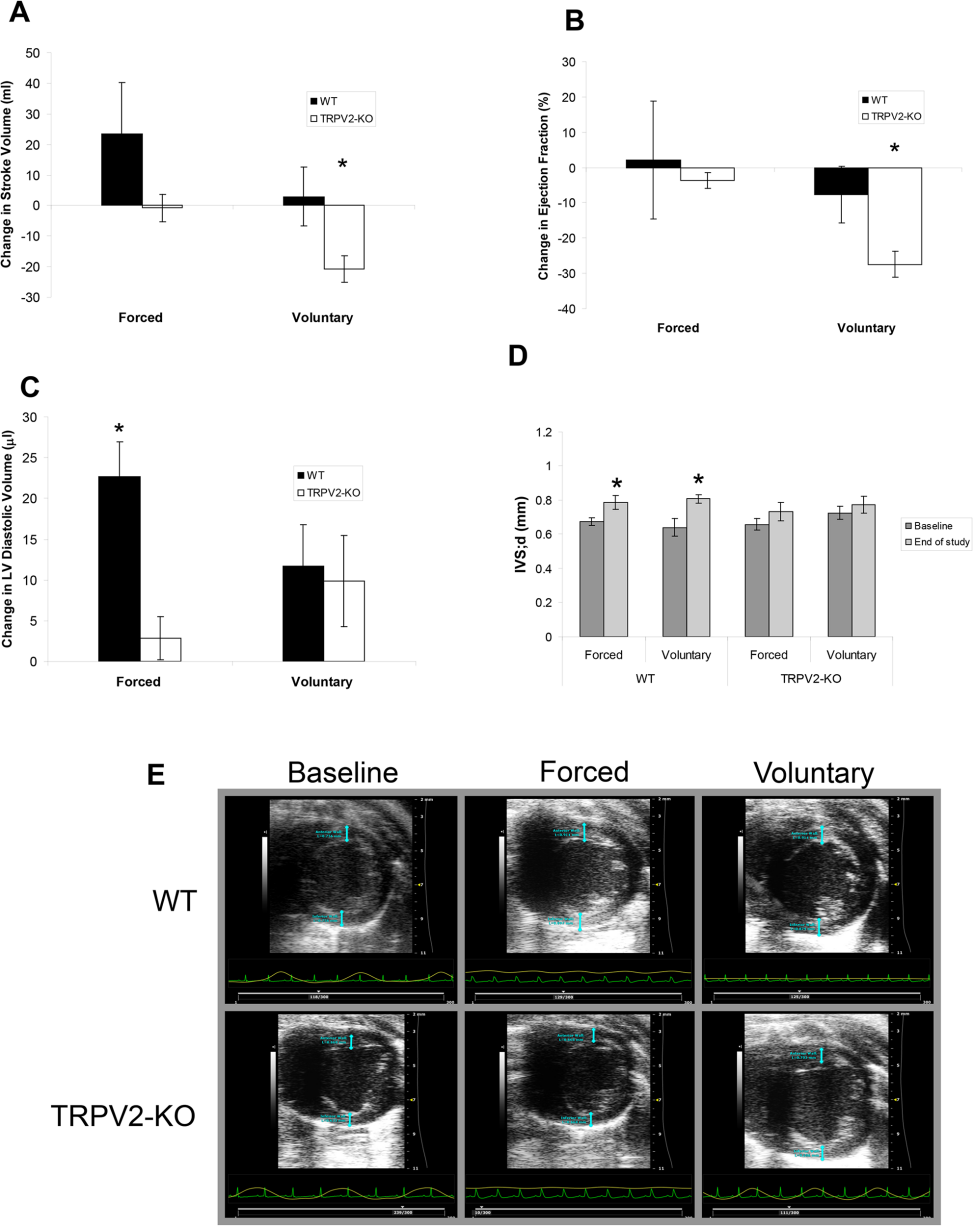
Echocardiographic parameters of WT and TRPV2-KO mice during forced and voluntary exercise. Change in (A) Stroke volume, (B) Ejection Fraction and (C) LV diastolic size in WT and TRPV2-KO mice exposed to forced and voluntary exercise. D. IVS;d measurements at baseline and after the exercise protocols. (*P<0.05, as compared to baseline). E. Representative SAX images for WT and TRPV2-KO mice at baseline, after forced exercise (4 weeks) and after voluntary exercise (8 weeks).

During all exercise studies, a longitudinal evaluation of cardiac function was performed via echocardiography. Under forced conditions, WT mice demonstrated an increased stroke volume, although there were no changes found for the TRPV2-KO mice. Under voluntary conditions, there was no change in SV for the WT mice, while a significant decrease in SV was shown for the TRPV2-KO mice (Fig 5A). There was no change in EF for the WT mice under either exercise condition or the forced TRPV2-KO mice, yet the voluntary exercise TRPV2-KO mice had a significant decrease in EF (Fig 5B). WT mice demonstrated an increase in left ventricular diastolic volume after forced exercise, though there were no changes found for the TRPV2-KO or WT after voluntary exercise (Fig 5C). Hypertrophy was noted via echocardiography by an increased IVS;d wall thickness in WT mice under both exercise conditions, with no changes seen in the TRPV2-KO (Fig 5D). The impaired baseline cardiac function of the TRPV2-KO mice was worsened by exercise, especially after voluntary exercise, suggesting that even though the mice were able to exercise, there were not able to generate a compensatory response. Representative short axis echocardiographic images show concentric hypertrophy in the WT mice after forced and voluntary exercise (Fig 5E).

#### Voluntary exercise affected myocyte size of TRPV2-KO mice

Measurements of myocyte area in H & E stained sections in WT mouse hearts increased after voluntary exercise, as compared to control with no exercise (418.23+/-23.02 μm^2^ vs. 273.43+/-28.14 μm^2^, P<0.01). Furthermore, the TRPV2-KO mice showed a decrease in myocyte size after voluntary exercise (290.26+/-20.48 μm^2^) in comparison to the both TRPV2-KO control (290.26+/-20.48) and WT voluntary exercise (P<0.01).

#### TRPV2 modulates the response to physiologic stress

**Fig 6:**
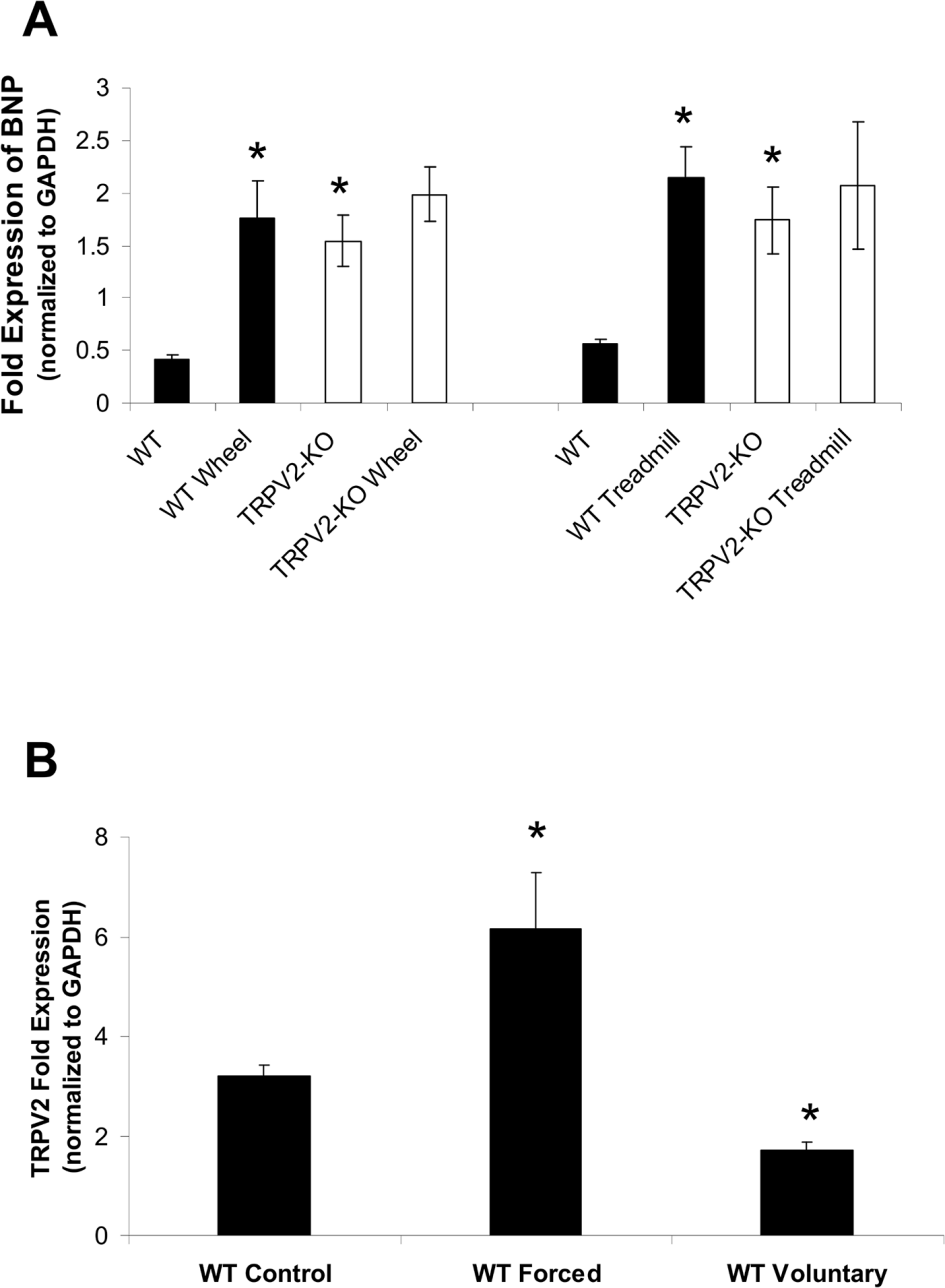
Gene expression patterns in WT and TRPV2-KO mice. (A) Brain natriuretic peptide (BNP) levels in WT and TRPV2-KO mice at control and after their respective exercise protocols. (B) Cardiac TRPV2 levels in WT mice after exercise. (*P<0.05, as compared to WT control).

BNP expression levels did increased after forced and voluntary exercise for the WT, but were already increased in the TRPV2-KO mice and did not increase further after exercise (Fig 6A). Importantly, TRPV2 levels as measured in WT mice decreased after voluntary exercise and increased after forced exercise (Fig 6B). These findings are suggestive of an important role for TRPV2 in mediating the cardiac response to physiologic stress.

## Discussion

The myocardial response to increased pressure in order to maintain cardiac performance was described by Grossman and others as far back as 1975 [26]. In the subsequent decades, the mechanisms via which these compensatory effects occur under physiologic conditions has been somewhat clarified and appear to involve complex and overlapping pathways that result in increased cardiac size, improved myocardial function and small but measurable changes in specific gene and protein expression [1,2,3]. Certain TRP channels, such as TRPV2 and TRP canonical 1 (TRPC1), are known to be stretch sensitive signaling mediators that are calcium permeable [27]. Recently, the TRPC1 channels in the myocardium have been shown to mediate hypertrophic signaling [28], while others have shown that TRPV2 channels play a role in the development of dilated cardiomyopathy [11] and in maintaining structural integrity of the heart [17], though this is the first time that the TRPV2 channels have been shown to mediate a cardiac response to exercise.

We had previously documented that the absence of TRPV2 results in decreased systolic function. In this study, we demonstrate that the channel not only modulates cardiac function, but also the hypertrophic response to stress. The TRPV2-KO mice demonstrate an impaired response to pressure overload and after voluntary exercise the myocyte size is decreased. This finding has important implications in the exercise capacity of the mice, as the mice exposed to voluntary running conditions were, after several weeks, able to exercise to a similar degree as their counterparts without an observable improvement in cardiac function.

Regarding the role that TRP channels in general may play in skeletal muscle, it has been previously documented that TRPC1 channels play a role in skeletal muscle function [29]. Specifically, TRPC1 was found to modulate Ca^2+^ influx in the skeletal muscle and help muscles maintain their force during sustained contractions. In contrast, we found no significant role for TRPV2 in skeletal muscle function. Interestingly, Zanou et al. did find that the skeletal myocytes of the TRPC1-KO mice were significantly smaller than the WT, not unlike our finding in cardiac myocytes, thus implying a role for these TRP channels in modulating striated muscle function and the development of hypertrophy.

We have previously published about the impaired cardiac function of the TRPV2-KO mouse [7]. In this study, we explore the effects of TRPV2 ablation on exercise capacity and ventricular remodeling. The TRPV2-KO mice were unable to complete the forced exercise protocol, and therefore did not show any changes in cardiac function. The reason behind their inability to run properly on the treadmill could have been due to other factors including neurologic or behavioral; however Park et al [15]. did not find any neurophysiological phenotype associated with the TRPV2-KO mice that may explain this finding. Furthermore, the TRPV2-KO mice were able to increase their time and distance when allowed to exercise voluntarily. It is important to note that the echocardiographic measurements indicated that the voluntary exercise actually exacerbated their cardiac dysfunction as demonstrated by a decrease in SV and EF, with lack of development of compensatory hypertrophy. This lack of compensation can potentially be explained by non-cardiac effects, though as we have previously published there is no significant vascular phenotype [7] and as presented in this paper there is also no obvious muscular-skeletal phenotype. In contrast, WT mice clearly had an increase in wall thickness, indicating hypertrophy and ventricular remodeling after forced and voluntary running.

Lastly, we found an increase in cardiac TRPV2 expression after forced exercise. When considered in light of the cardiac impairment observed in the TRPV2-KO mice after voluntary exercise, it strongly implies a dynamic role for the channel as hypertrophy develops and the myocardium response to stressors. These findings are consistent with our previously published data, as well as the data from Katanoska [17] and Iwata [11] that demonstrate a crucial role for TRPV2 in regulating calcium influx and the development of cardiomyopathy under baseline and pathological conditions and add to this wealth of information data regarding the channel and its important effects under physiologic stress conditions.

Future studies will focus on the time frame of the changes in expression of TRPV2 as well as the potential differences between our TRPV2- whole body, pore deletion KO model and other models that use cardiac specific whole channel KO mice.

### Clinical Perspectives

The development of cardiomyocyte hypertrophy in response to exercise-induced stress is an important adaptive mechanism that permits acute and chronic changes to occur to the heart in order to maintain cardiac output. In this study, we demonstrate that TRPV2 plays a crucial role in modulating this response. In future studies, we will attempt to elucidate if this blunted result is also present under pathologic conditions and therefore may serve as a target for the prevention of hypertrophy as the compensatory mechanism becomes maladaptive and leads to development of left ventricular hypertrophy and heart failure.

### Conclusion

TRPV2 channels modulate the cardiac response to exercise stress.

## Supporting Information

S1 Dataset qRT-PCR data for Fig 1. (XLS)

S2 Dataset Raw data for Fig 2A. (XLS)

S3 Dataset Raw data for Fig 3A–3D. (XLS)

S4 Dataset Raw data for Fig 4A–4D. (XLS)

S5 Dataset Raw data for Fig 5A–5D. (XLS)

S6 Dataset Raw data for Fig 6A & 6B. (XLS)
